# Polymorphisms of the *GCLC* Gene Are Novel Genetic Markers for Susceptibility to Psoriasis Associated with Alcohol Abuse and Cigarette Smoking

**DOI:** 10.3390/life13061316

**Published:** 2023-06-02

**Authors:** Ekaterina Efanova, Olga Bushueva, Roman Saranyuk, Anna Surovtseva, Mikhail Churnosov, Maria Solodilova, Alexey Polonikov

**Affiliations:** 1 Medvenka Central District Hospital, 68 Sovetskaya Street, 307030 Kursk, Russia; 2 Laboratory of Genomic Research, Research Institute for Genetic and Molecular Epidemiology, Kursk State Medical University, 18 Yamskaya Street, 305041 Kursk, Russia; olga.bushueva@inbox.ru (O.B.);; 3 Department of Biology, Medical Genetics and Ecology, Kursk State Medical University, 3 Karl Marx Street, 305041 Kursk, Russia; 4 Center for Medical Examinations and Prevention, 2 Leninsky Komsomol Avenue, 305026 Kursk, Russia; 5 Department of Medical Biological Disciplines, Belgorod State University, 85 Pobedy Street, 308015 Belgorod, Russia; churnosov@bsu.edu.ru; 6 Laboratory of Statistical Genetics and Bioinformatics, Research Institute for Genetic and Molecular Epidemiology, Kursk State Medical University, 18 Yamskaya Street, 305041 Kursk, Russia

**Keywords:** psoriasis, genetic susceptibility, oxidative stress, glutathione, glutamate cysteine ligase, *GCLC*, single nucleotide polymorphism, cigarette smoking, alcohol abuse, gene–environment interactions

## Abstract

The aim of this pilot study was to investigate whether single nucleotide polymorphisms (SNP) in the gene encoding the catalytic subunit of glutamate cysteine ligase (*GCLC*) are associated with the risk and clinical features of psoriasis. A total of 944 unrelated individuals, including 474 patients with a diagnosis of psoriasis and 470 healthy controls, were recruited for the study. Six common SNPs in the *GCLC* gene were genotyped using the MassArray-4 system. Polymorphisms rs648595 (OR = 0.56, 95% CI 0.35–0.90; P_perm_ = 0.017) and rs2397147 (OR = 0.54, 95% CI 0.30–0.98; P_perm_ = 0.05) were associated with susceptibility to psoriasis in males. In the male group, diplotype rs2397147-C/C × rs17883901-G/G was associated with a decreased risk of psoriasis (FDR-adjusted *p* = 0.014), whereas diplotype rs6933870-G/G × rs17883901-G/G (FDR-adjusted *p* = 0.045) showed an association with an increased disease risk in females. The joint effects of SNPs with tobacco smoking (rs648595 and rs17883901) and alcohol abuse (rs648595 and rs542914) on psoriasis risk were observed (P_perm_ ≤ 0.05). We also found multiple sex-independent associations between *GCLC* gene polymorphisms and various clinical features such as earlier disease onset, the psoriatic triad, and specific localizations of skin lesions. The present study is the first to show that polymorphisms of the *GCLC* gene are significantly associated with the risk of psoriasis and related to its clinical features.

## 1. Introduction

Psoriasis is a chronic immune-inflammatory-mediated dermatosis characterized by thickened, scaly erythema or plaques [1,2]. Psoriasis is recognized by the World Health Organization as a serious non-communicable disease [3]. Clinical variants of the disease include psoriasis vulgaris, arthritis, and pustular and erythrodermic types; however, psoriasis vulgaris is the most common form, accounting for about 90% of cases and affecting 3% of Caucasians [4]. A study by Kubanov and co-workers demonstrated a substantial disease burden on psoriasis patients in Russia [5].

The etiology and pathogenesis of psoriasis remain mysteries, making the disease’s management more challenging [6]. Psoriasis is characterized by sustained inflammation, which results in uncontrolled keratinocyte proliferation and defective differentiation [7]. Psoriatic inflammation is caused and maintained by disruptions in innate and adaptive cutaneous immune responses [6,8], which coexist with autoinflammatory perpetuation or T-cell-driven autoimmune reactions [7]. The overlap of autoimmune and autoinflammatory mechanisms in the pathogenesis of psoriasis has led to the development of biological therapy for the disease. However, despite the fact that targeted therapies focusing on the inhibition of cytokines such as IL-23 and IL-17 showed high clinical efficacy, psoriasis remains an incurable disease [7].

Psoriasis is known as a complex multifactorial disease for which development is determined by the interaction between genetic, environmental, and epigenetic factors [9,10,11]. Linkage analysis, an effective method to identify the chromosomal location of disease genes, has discovered nine separate genomic regions known as psoriasis susceptibility regions (PSORS1-9) comprising many genetic variants, a part of which has been fine-mapped as disease-linked loci [11,12]. Progress in the development of high-throughput genotyping technologies enabled the implementation of genome-wide association studies (GWAS), a research approach in which large case–control cohorts are genotyped for tens of thousands of single nucleotide polymorphisms (SNPs) across the genome [11]. According to the GWAS catalog (https://www.ebi.ac.uk/gwas/home, accessed on 29 April 2023), 57 GWASs have been conducted so far to unravel the genetic background of psoriasis in different populations around the world, and 946 SNPs have been identified as loci associated with disease susceptibility or severity and those influencing the efficacy of anti-psoriatic therapy. Nevertheless, despite considerable genetic research and achievements, the etiology of psoriasis and its primary molecular mechanisms remain elusive.

It has been argued that the increased production of reactive oxygen species (ROS) and a decreased antioxidant defense leading to the activation of oxidative stress are involved in the pathogenesis of psoriasis and influence disease duration and severity [13,14,15,16]. Despite the fact that the important role of oxidative stress in the etiopathogenesis of psoriasis remains undisputable after decades of research, a limited number of studies have been undertaken so far to assess whether genetic variation in antioxidant defense enzymes contributes to psoriasis susceptibility. A larger portion of the studies looked for the link between psoriasis risk and genetic polymorphisms of glutathione-S-transferases [17,18,19,20], enzymes catalyzing the conjugation of reduced glutathione (GSH) to xenobiotic compounds for their detoxification.

Glutathione is a low-molecular-weight thiol, a tripeptide consisting of glutamate, cysteine, and glycine, which plays a major role in maintaining intracellular redox balance and antioxidant defense [21]. It is involved in many crucial biological functions, such as xenobiotic detoxification, maintaining mitochondrial function, the modulation of cell proliferation, wound healing, and the inhibition of apoptosis [21,22]. Furthermore, glutathione is utilized as a cofactor by glutathione peroxidases and glutathione S-transferases for the glutathionylation of selected proteins and toxic substance conjugation. GSH is also required for the maturation of cytosolic iron–sulfur proteins, which are essential for cell viability and involved in the maintenance of DNA metabolism, genome integrity, protein translation, and other critical biological functions [22,23]. It is important to note that glutathione is involved in the skin metabolic clearance system [24], protects DNA and mitochondria from oxidative damage, and ensures the survival of keratinocytes in normal and wounded skin [25]. Glutathione deficiency is well known to be associated with an increased susceptibility to oxidative stress, a pathological condition implicated in the pathogenesis of psoriasis [26], and, therefore, we can suggest that oxidative stress may be responsible for the modulation of inflammatory and autoimmune mechanisms underlying the diseases [27,28]. Despite the obvious importance of glutathione in skin metabolism, existing research data in psoriasis on the roles of genes encoding enzymes involved in glutathione metabolism, primarily glutamate cysteine ligase, an enzyme catalyzing the initial rate-limiting step of GSH biosynthesis [29], are surprisingly absent. We propose that genetic polymorphisms of glutamate cysteine ligase may explain inter-individual differences in glutathione biosynthesis and influence the risk of psoriasis, making SNPs attractive markers for testing disease susceptibility. Therefore, the purpose of our pilot study was to investigate whether common polymorphisms at the gene encoding the catalytic subunit of glutamate cysteine ligase (*GCLC*) are associated with the risk and clinical features of psoriasis.

## 2. Materials and Methods

### 2.1. Study Participants and Clinical Examination

Informed consent was signed by all subjects involved in this study. The protocol of the present study was approved by the Ethical Review Committee of Kursk State Medical University (protocol No. 8, 13.11.2017). A total of 944 unrelated individuals of European descent (predominantly Russians), including 474 patients with a diagnosis of psoriasis and 470 healthy controls, were used for this study. The enrollment of patients with psoriasis was conducted in Medvenka Central District Hospital (Kursk region), the Center for Medical Examinations and Prevention (Kursk), and Kursk Regional Multidisciplinary Clinical Hospital in a period between September 2018 and December 2021. The control group of subjects without chronic diseases was recruited from our previous studies [30,31,32]. The diagnosis of psoriasis was verified by qualified dermatologists based on the typical clinical picture of skin rashes and their localization [6]. The study included patients with classic plaque psoriasis; palmoplantar, seborrheic, and scalp psoriasis; the von Zumbusch type of generalized pustular psoriasis; inverse psoriasis; guttate psoriasis; and erythrodermic psoriasis, as well as psoriasis comorbidities such as psoriatic arthritis and onychodystrophy [33]. The Psoriasis Area and Severity Index (PASI) was used for the clinical assessment of the severity of the course of psoriasis [34]. Enrolled patients did not suffer from chronic infectious diseases, including HIV and hepatitis, and did not have severe chronic conditions that manifested before psoriasis. Patients who were receiving biologic therapy at the time of the recruitment and pregnant women were not included in the study. Study participants completed a validated doctor-administered questionnaire [35] to assess risk factors for psoriasis, such as cigarette smoking [36] and alcohol consumption [37]. Information on smoking status (ever/never) was available from all psoriatic patients and healthy subjects. Data on alcohol intake were available from all patients with psoriasis and only 220 individuals from the control group. Alcohol intake habits were assessed by the number of drinks consumed per week, as described previously [38,39]. Briefly, according to the reported frequency of alcohol intake, study individuals were categorized into two groups: (1) subjects who consumed alcohol 1 to 2 days a month or less and (2) those drinking alcohol 1 or more days a week. The second group was considered alcohol abusers.

### 2.2. Selection of Single Nucleotide Polymorphisms (SNPs)

GCLC is a catalytic subunit of glutamate-cysteine ligase and is the first rate-limiting enzyme of glutathione synthesis [29]. Six common (minor allele frequency ≥ 5%) SNPs, including rs524553, rs542914, rs648595, rs6933870, rs2397147, and rs17883901, of the *GCLC* gene were selected for the study according to the functional properties of the polymorphisms (the presence of eQTL, expression quantitative trait loci, in the skin from GTEx portal, https://gtexportal.org) and linkage disequilibrium (r2 ≥ 0.8) between them (HapMap data, European population). Candidate Gene SNP Selection (GenePipe) at the SNPinfo Web Server (https://snpinfo.niehs.nih.gov/snpinfo/selegene.html (accessed on 25 April 2021)) was used for SNP selection.

### 2.3. Genetic Analysis

Venous blood samples were collected from the cubital vein of study subjects into EDTA-coated tubes and immediately frozen and stored at −20 °C until processed. Total DNA was purified by the standard phenol/chloroform extraction and ethanol precipitation. Genotyping of the SNPs was performed with the MALDI-TOF mass spectrometry iPLEX platform on the MassArray-4 system (Agena Bioscience, Inc., San Diego, CA, USA). Primer sequences used for genotyping are available upon request. To guarantee quality control, 5% of DNA samples were genotyped in duplicates while researchers were blind to the case–control status. The concordance rate of the control genotyping was >99%. Genetic investigations were carried out at the Research Institute for Genetic and Molecular Epidemiology of Kursk State Medical University (Kursk, Russia).

### 2.4. Statistical Analysis

Statistical power was estimated using the GAS power calculator (https://csg.sph.umich.edu/abecasis/gas_power_calculator/, accessed on 21 May 2022). It has been estimated that we could detect a genotype relative risk (GRR) of 1.30–1.45 with 82–98% power in the overall analysis (474 cases and 470 controls) and a GRR of 1.40–1.5 with 76–83% power in the analysis of groups stratified by sex/risk factors at α = 0.05. Fisher’s exact test was used to assess the distribution of genotype frequencies according to the Hardy–Weinberg equilibrium (HWE). Allele and genotype frequencies in the study groups and their associations with the risk of psoriasis were analyzed using the PLINK software v.1.9 [40]. Logistic regression analysis was used to evaluate the associations of *GCLC* gene polymorphisms with the risk of psoriasis and binary clinical phenotypes. The crude odds ratio (OR) and 95 percent confidence intervals (95% CI) were calculated to assess SNP–phenotype associations phenotype associations. Associations of SNPs with continuous phenotypes were evaluated with linear regression analysis, with estimation of differences in mean between genotypes and 95% CI using the SNPstats software (https://www.snpstats.net/start.htm, accessed on 12 April 2023). For SNP–disease associations, allelic, recessive, dominant, and log-additive genetic models were evaluated. Haplotype analysis and visualization of the haplotypic structure of the *GCLC* gene were performed by the Haploview software, v.4.2 [41]. *p*-values (P_perm_) for allele/genotype/haplotype associations were estimated via adaptive permutations using PLINK and Haploview. Gene–environment interactions were analyzed in groups stratified by risk factors such as cigarette smoking and alcohol abuse. Replication of associations between *GCLC* gene polymorphisms and psoriasis was performed using the Gene ATLAS database of the UK Biobank (http://geneatlas.roslin.ed.ac.uk (accessed on 17 January 2023)). Associations of pairwise genotype combinations (diplotypes) with the risk of psoriasis were estimated by the chi-squared test and adjusted for multiple comparisons by the false discovery rate (FDR) procedure (False Discovery Rate Online Calculator, https://tools.carbocation.com/FDR, accessed on 9 April 2023).

## 3. Results

### 3.1. Baseline and Clinical Characteristics of the Study Patients

The baseline and clinical characteristics of the study patients are listed in Table 1. The group of patients with psoriasis was matched to the control group for sex (*p* = 0.30). The psoriasis patients were more than ten years younger than the healthy subjects. The duration of psoriasis was 10 (4–21) years. The mean age of disease onset was 27 (18–40) years old. The number of smokers in each group was about equal. However, the number of subjects abusing alcohol in the patient group was seven times higher than in the control group (*p* < 0.0001). The psoriatic triad was diagnosed in 54.4% of patients. Most often, psoriatic rashes in patients were observed in the upper (80.0%) and lower (57.4%) extremities, the head (47.9%), and the trunk (33.08%), which is typical for psoriasis.

The most prevalent comorbidities among psoriasis patients were hypertension (22.6%), chronic renal (6.4%), and gastrointestinal (7.0%) diseases.

### 3.2. Association of GCLC Gene Polymorphisms with the Risk of Psoriasis

Genotype frequencies for five polymorphisms of the *GCLC* gene satisfied the Hardy–Weinberg equilibrium in both cases and controls. Only one SNP, rs17883901, showed a deviation from the HWE in both groups (*p* = 0.001). We analyzed associations between the *GCLC* gene polymorphisms and the risk of psoriasis in entire groups and groups stratified by sex. Table 2 shows a summary of associations between *GCLC* gene polymorphisms and psoriasis risk in the entire and sex-stratified groups. Allelic, additive, dominant, and recessive genetic models of SNP–disease associations were evaluated, and *p*-values (P_perm_) were assessed using adaptive permutation tests. The most significant Pperm was considered to be the selected genetic model of SNP–disease associations.

The genotype and allele frequencies of the *GCLC* gene in healthy controls and patients with psoriasis, along with the most significant P_perm_ of the SNP–disease associations, are reported in Table 3. As can be seen from Table 3, none of the polymorphisms showed an association with the risk of psoriasis as analyzed in the entire group of patients. However, the sex-stratified analysis detected that SNPs rs648595 (OR = 0.56, 95% CI 0.35–0.90; P_perm_ = 0.017, recessive model) and rs2397147 (OR = 0.54, 95% CI 0.30–0.98; P_perm_ = 0.05, recessive model) of the *GCLC* gene were associated with susceptibility to psoriasis in males. None of the polymorphisms was significantly associated with the risk of psoriasis in females.

### 3.3. Joint Effects of GCLC Gene Polymorphisms on the Risk of Psoriasis

**Figure 1:**
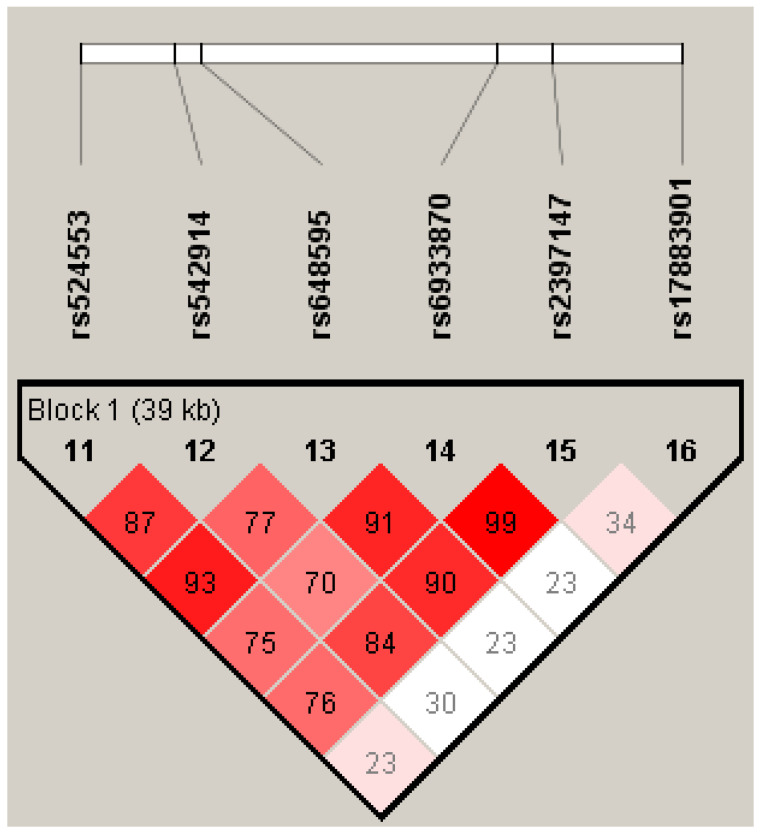
Linkage disequilibrium (LD) plot of the *GCLC* gene generated by the Haploview software, v.4.2. Lewontin’s standardized coefficient D’ values serve as a means to represent LD. The magnitude and significance of pairwise LD are shown by shading, with a red-to-white gradient showing higher-to-lower LD values.

The joint effects of *GCLC* gene polymorphisms on psoriasis risk were evaluated via haplotype and diplotype analyses. The *GCLC* haplotypes and their association with psoriasis risk in the entire and sex-stratified groups are shown in Table 4. Four common haplotypes of *GCLC* (H1–H4) with a frequency of more than 5% were identified in the study groups. The rare haplotype H12, with a frequency of 1%, was detected only in females. Figure 1 shows the linkage disequilibrium plot of the *GCLC* gene generated by the Haploview software. The polymorphism rs17883901 was not linked to any of the other studied SNPs in the *GCLC* gene. As can be seen from Table 4, none of the haplotypes was meaningfully associated with the risk of psoriasis, both in the entire and sex-stratified groups (P_perm_ > 0.05).

The results of the diplotype analysis are shown in Table 5. In the entire group, genotype combinations such as rs542914-C/C × rs648595-G/T (FDR-adjusted *p* = 0.03) and rs648595-G/G × rs6933870-C/G (FDR-adjusted *p* = 0.016) of *GCLC* showed associations with an increased and decreased risk of psoriasis, respectively. In the male group, diplotype rs2397147-C/C × rs17883901-G/G was associated with a decreased risk of psoriasis (FDR-adjusted *p* = 0.014), whereas diplotype rs6933870-G/G × rs17883901-G/G (FDR-adjusted *p* = 0.045) showed an association with an increased disease risk in females. The remaining six diplotypes associated with disease risk in males did not reach statistical significance after adjusting for multiple tests.

### 3.4. Gene–Environment Interactions and Psoriasis Risk

Since psoriasis is a multifactorial disease, it appears important to investigate the joint influence of environmental risk factors and gene polymorphisms on disease development. Two risk factors, such as cigarette smoking and alcohol abuse, were used for the analysis of gene–environment interactions in psoriasis. Table 6 shows a summary of associations between *GCLC* gene polymorphisms and psoriasis risk in groups stratified by cigarette smoking and alcohol abuse habits. We found that SNP rs648595 is associated with the risk of psoriasis in cigarette smokers (OR = 0.55, 95% CI 0.31–0.99; P_perm_ = 0.049, recessive model), whereas no association of this polymorphism was seen in non-smokers (OR = 0.88, 95% CI 0.59–1.31; P_perm_ = 0.52, recessive model). In contrast, SNP rs17883901 showed an association with the risk of psoriasis in non-smokers (OR = 0.22, 95% CI 0.02–1.97; *p* = 0.14; P_perm_ = 0.002, recessive model), whereas no association with this variant was observed in smoker subjects (OR = 0.89, 95% CI 0.11–5.90; *p* = 0.84; P_perm_ = 0.99, recessive model). Notably, polymorphisms rs542914 (OR = 0.57, 95% CI 0.36–0.90; P_perm_ = 0.015, recessive model) and rs648595 (OR = 0.60, 95% CI 0.39–0.92; P_perm_ = 0.03, recessive model) of *GCLC* were associated with a decreased risk of psoriasis in non-drinkers of alcohol.

However, no protective effects of these SNPs against the risk of psoriasis were identified in alcohol abusers (*p* > 0.05).

### 3.5. Replication of Associations between GCLC Gene Polymorphisms and Psoriasis Risk in a Population of UK Biobank

It is stated that replication helps ensure that a genotype–phenotype relationship discovered in an original study represents a credible association and is not a chance finding or an artifact due to uncontrolled biases [42,43]. Therefore, we performed a replication analysis of associations between the studied *GCLC* gene polymorphisms and psoriasis susceptibility in two large populations from the UK Biobank. Table 7 shows the results of replication analysis to confirm associations between the studied polymorphisms of the *GCLC* gene and psoriasis risk in a population of the UK Biobank. It has been revealed that two SNPs of *GCLC*, rs6933870 (*p* = 0.063) and rs2397147 (*p* = 0.057), showed a clear tendency in their association with the risk of psoriasis in one of the UK cohorts. Formally, we cannot conclude that the *GCLC* gene polymorphisms we studied have been replicated in an independent population. The non-replication of SNP–disease associations might be in part explained by inter-population genetic differences, and this issue has been proposed to be readily resolved by the use of a gene-based approach rather than either an SNP-based or a haplotype-based approach [42,44]. Pursuing this proposal, we performed an association analysis of psoriasis with all SNPs of the GCLC gene genotyped in the UK Biobank cohorts. As a result (Table 8), 75 and 21 SNPs of the GCLC gene in the first and second UK Biobank cohorts, respectively, have been found to be associated with the risk of psoriasis at a *p*-value ≤ 0.05. Two polymorphisms of GCLC, rs547541077 (*p* = 0.004) and rs7764361 (*p* = 0.039), were associated with psoriasis risk in both cohorts.

### 3.6. Association of GCLC Gene Polymorphisms with Clinical Features of Psoriasis

**Figure 2:**
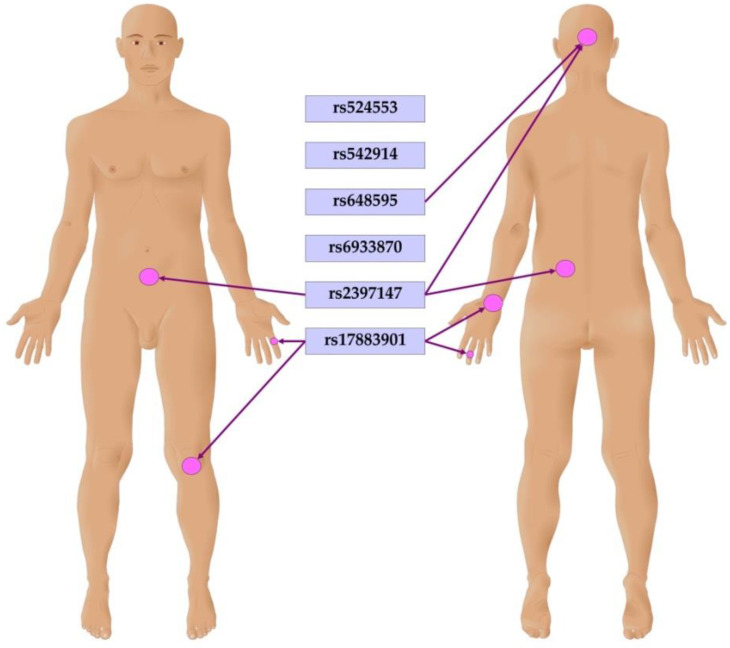
Associations of *GCLC* gene polymorphisms with psoriatic lesions on the body.

The associations of *GCLC* gene polymorphisms with clinical manifestations of psoriasis were analyzed and adjusted for sex. It has been revealed that a carriage of genotypes rs542914CA and AA of *GCLC* was positively associated with the psoriatic triad (OR = 1.72, 95% CI 1.18–2.51; *p* = 0.005). An earlier onset of psoriasis was associated with the effects of SNPs rs648595 (difference −2.04, 95% CI −3.67–−0.40, *p* = 0.015) and rs6933870 (difference −1.73, 95%CI −3.36–−0.10, *p* = 0.038). The carriage of genotype rs524553TT of *GCLC* was found to be associated with more frequent flare-ups of psoriasis (difference 0.67, 95% CI 0.01–1.33, *p* = 0.047). Polymorphisms have been found to be associated with psoriasis localization features. Figure 2 summarizes the findings of the analysis. SNP rs648595 showed association with scalp psoriasis (OR = 1.32, 95% CI 1.01–1.74; *p* = 0.04, log-additive genetic model). Polymorphisms rs648595 (difference 0.17, 95% CI 0.00–0.35, *p* = 0.048, additive genetic model) and rs2397147 (difference 0.27, 95% CI 0.03–0.50, *p* = 0.025, overdominant genetic model) of *GCLC* were associated with an increased area of skin lesions on the scalp. In addition, genotypes rs2397147TC and C/C were associated with increased infiltration (difference 0.23, 95% CI 0.03–0.43, *p* = 0.023) and peeling (difference 0.22, 95% CI 0.03–0.42, *p* = 0.026) of psoriatic lesions on the trunk. Genotype rs524553CT was also associated with increased infiltration (difference 0.22, 95% CI 0.01–0.42, *p* = 0.037) and peeling (difference 0.22, 95% CI 0.02–0.43, *p* = 0.029) of psoriatic lesions on the trunk. The polymorphism rs17883901 of GCLC was found to be associated with psoriasis on the knees (OR = 2.34, 95% CI 1.20–4.58; *p* = 0.019, additive genetic model). Moreover, genotype rs17883901AA was associated with psoriasis on the wrist (OR = 31.25, 95% CI 2.68–364.40; *p* = 0.007) and fingers (OR = 13.99, 95% CI 1.25–157.15; *p* = 0.03, recessive model). Interestingly, genotypes rs648595 GT and GG were also found to be associated with type 2 diabetes in patients with psoriasis (OR = 2.80, 95% CI 1.06–7.37; *p* = 0.021). Notably, all the observed associations with clinical features occurred regardless of sex.

## 4. Discussion

Since the skin is frequently exposed to environmental insults such as ultraviolet irradiation, exposure to toxic chemicals, or mechanical injury causing oxidative or chemical stress, one of the principal physiologic roles of the skin is as a robust barrier against xenobiotics and free radicals for their metabolic elimination and detoxification [25,45,46]. For promoting these functions, human skin possesses a significant potential for phase II metabolism via multiple reactions including glutathione conjugation [45], and, therefore, the cytoprotective effects of GSH are likely to be of importance in this tissue. Experimental studies by Telorack and co-workers [25] have revealed that knockout mice with keratinocyte-specific deficiency in glutamate cysteine ligase showed a strong reduction in the viability of cell culture in vitro and in the skin in vivo. Furthermore, the authors observed that keratinocytes in glutathione-deficient mice died from apoptosis, ferroptosis, and necroptosis, and the increased cell death was attributed to increased levels of reactive oxygen and nitrogen species, causing DNA and mitochondrial damage [25]. This important research demonstrates the epidermis’s exceptional antioxidant capability (especially with glutathione), which ensures skin integrity and effective wound healing. A deficiency of skin glutathione may contribute to psoriasis development. Genetic polymorphisms of glutamate cysteine ligase that are correlated with a decrease in GCLC mRNA and protein expression, enzyme activity, and GSH content [47,48,49,50,51] represent attractive markers for studying the molecular mechanisms of psoriasis. Polymorphisms of the GCLC gene have been found to be associated with the risk of cardiometabolic diseases such as coronary artery disease [52,53], ischemic stroke [54], type 1 [55,56] and type 2 [51] diabetes mellitus, polycystic ovary syndrome [57], and nonalcoholic fatty liver disease [58], as well as other multifactorial disorders such as bronchial asthma [59], pulmonary tuberculosis [60], and colorectal cancer [61]. However, no studies have been designed so far to investigate the role of GCLC gene polymorphisms in psoriasis susceptibility.

The present study is the first to show that polymorphisms of the *GCLC* gene are significantly associated with the risk of psoriasis and related to its clinical features. Two SNPs, rs648595 and rs2397147, were found to be associated with a decreased risk of psoriasis in males, suggesting sexual dimorphism in the relationship between the gene variation and susceptibility to psoriasis. Sexual dimorphism was also seen in associations between *GCLC* diplotypes and disease risk: rs2397147-C/C × rs17883901-G/G was associated with a decreased risk of psoriasis in males, whereas diplotype rs6933870-G/G × rs17883901-G/G showed an association with an increased disease risk in females. These findings were not surprising because gender differences in psoriasis risk and severity have become a discussable issue among dermatologists in the last few years [62,63].

Notably, sexual dimorphism has also been demonstrated in some genetic association studies on skin disease such as atopic dermatitis [64]. Environmental risk factors such as cigarette smoking and alcohol abuse may explain the mechanisms by which sexual dimorphism determines susceptibility to psoriasis [36,37,65]. We investigated the effect of *GCLC* gene polymorphisms on psoriasis risk depending on these environmental risk factors in the studied population. We found that polymorphism rs648595 is associated with the risk of psoriasis in cigarette smokers exclusively. Another SNP of *GCLC*, rs17883901, was associated with the risk of psoriasis only in non-smokers. Furthermore, polymorphisms rs542914 and rs648595 were found to be associated with a decreased risk of psoriasis in non-drinkers of alcohol, whereas no protective effects of these SNPs against disease risk were seen in subjects who were alcohol abusers.

The present study revealed sex-independent associations between GCLC gene polymorphisms and some clinical features such as the psoriatic triad, earlier onset, and more frequent flare-ups of disease, as well as localizations of psoriatic lesions. The last finding suggests that there are area-specific genetic effects of the studied polymorphisms of the *GCLC* gene that may be attributed to inter-individual differences in gene expression and, therefore, rates in glutathione biosynthesis by the skin from different body areas, as was demonstrated with regard to the rate of glutathione conjugation in different organs [66]. It is also known that the levels of glutathione may vary in sun-exposed and sun-protected areas [67], suggesting that UV exposure may impact glutathione biosynthesis in the skin.

The replication analysis in the UK Biobank cohorts showed a non-significant but clear association between rs6933870 and rs2397147 and psoriasis risk, suggesting that inter-population genetic differences may explain the non-replication of SNP–disease relationships. When we analyzed the associations between psoriasis and all the SNPs of the *GCLC* gene genotyped in the same cohorts, more than 70 polymorphisms were associated with disease risk, meaning that different SNPs may contribute to disease susceptibility in different ethnicities.

The functional annotation of some polymorphisms of the *GCLC* gene was performed in our previous study [54]. In particular, we found that allele rs648595G (this SNP showed the most significant association with psoriasis) is associated with a decreased expression of *GCLC* in blood, non-sun-exposed suprapubic skin, and sun-exposed lower leg skin. This SNP has regulatory potential and is located in transcription factor (TF)-binding or DNase hypersensitivity sites [54]. As predicted by HaploReg v4.2 tools, the rs648595 polymorphism is located within the TF-binding site for transcription factor AP-1 (activator protein 1), which is known to control gene expression in response to various stimuli such as cytokines, growth hormones, stress, and infections [68]. In the liver, SNP rs648595 is enriched with enhancer (H3K4me1 and H3K27ac) and promotor (H3K4me3 and H3K9ac) histone marks that regulate the transcriptional activity. In particular, H3K4me1 is a dynamic modification that was specifically found to mark both active and primed enhancers [69]. Enhancers bearing the H3K4me1 mark were found to be poised for activation in response to external stimuli [70]. H3K4me3 was found to promote rapid gene activation [71]. H3K9ac co-occurs highly with H3K14ac and H3K4me3 histone marks associated with active gene promoters [72]. Taken together, the epigenetic data clearly show that the polymorphism rs648595 of the *GCLC* gene represents an important genetic variant capable of activating gene expression in the liver.

Our findings of gene–environment interactions indicate that risk factors such as cigarette smoking and alcohol abuse can modify the associations between *GCLC* gene polymorphisms and the risk of psoriasis (Table 6). It is known that reduced glutathione plays an important role in ethanol detoxification, and acute ethanol administration was found to deplete GSH in the liver and other organs [73]. The leveling of the protective effects of the rs648595 and rs542914 polymorphisms in chronic alcohol abusers appears to be explained by the fact that persistent ethanol intake may diminish the endogenous pool of glutathione [74,75]. Meanwhile, an in vitro study by Kimura and co-workers [76] has revealed that primary human hepatocytes treated with 100 and 200 mM of ethanol showed the induction of *GCLC* gene expression via the activation of the NF-κB pathway. Tobacco smoking is also well known to deplete glutathione [77,78,79]. Thus, our study supports the causative roles of tobacco smoking and alcohol abuse in the development of psoriasis, and the negative effects of these environmental factors eliminate the protective role of polymorphisms of the *GCLC* gene against disease risk.

Sexual dimorphism in the discovered associations of *GCLC* gene polymorphisms with psoriasis risk is apparently attributed to differences in environmental exposures (i.e., smoking and alcohol abuse) between sexes. Considering an important role of oxidative stress in the pathogenesis of psoriasis [13,14,15,16], the mechanisms by which glutathione exerts protective effects against disease risk can be explained by the key role of glutathione in detoxifying ROS and environmental toxicants, penetrating and generating in the skin. However, the role of glutathione in psoriasis pathogenesis is not limited to protecting skin from oxidative damage. GSH is also involved in the regulation of cell proliferation, wound healing, and the inhibition of apoptotic pathways [21,22]. Furthermore, glutathione plays an important role in the regulation of the immune system and inflammation, two faces of the same biological coin [80]. Glutathione possesses a wide range of effects on the immune system, either activating or suppressing the immune response to control inflammation. In particular, reduced glutathione is required for the control of innate and adaptive immunological processes such as T-lymphocyte proliferation, the phagocytic activity of polymorphonuclear neutrophils, and dendritic cell functions, as well as antigen presentation by antigen-presenting cells [80,81,82]. Changes in glutathione concentrations may be critical in many autoimmune disease disorders, including psoriasis [83]. In particular, glutathione may suppress the immune reaction in mice with allergic contact dermatitis [84], inhibit the production of inflammatory cytokines, and maintain the adequate production of interferon-gamma by dendritic cells [80].

Our study has several limitations. Since our study was the first to investigate the contribution of *GCLC* gene polymorphisms to psoriasis risk in relatively small groups of patients, further studies in populations with a larger sample size are required to replicate the observed associations. The relatively small number of subjects in the study groups did not allow the analysis of the joint effects of *GCLC* gene polymorphisms and environmental risk factors (smoking and alcohol abuse) separately in males and females to obtain estimates of sex-specific gene–environment interactions contributing to psoriasis susceptibility. Since the studied polymorphisms of the *GCLC* gene are located in noncoding regions, their phenotypic effects should be interpreted with caution because no investigations were conducted to assess gene expression in skin biopsies from study patients. Further genetic association studies are recommended to follow the gene-based approach to look for the link between psoriasis and a wider spectrum of polymorphisms in the *GCLC* gene. Following this approach, nevertheless, it should be taken into account that SNPs might be characterized by weak or moderate phenotypic effects that cannot be reproduced in independent populations given their genetic heterogeneity in minor allele frequencies and linkage disequilibrium between the loci [85,86]. Importantly, some studies have recently reported genetic differences in glutathione metabolism between races or ethnicities [47,87].

## 5. Conclusions

The present study demonstrated, for the first time, that polymorphisms in the gene encoding the catalytic subunit of glutamate cysteine ligase represent novel genetic markers for susceptibility to psoriasis. The phenotypic effects of *GCLC* polymorphisms on psoriasis risk are modified by tobacco smoking and alcohol abuse, which are known environmental factors that increase disease risk. The *GCLC* gene may contribute to the pathogenesis of psoriasis via the diminished biosynthesis of glutathione in both the liver and skin, where GSH regulates a plethora of cellular processes such as redox homeostasis, the detoxification of xenobiotics, innate and adaptive immune functions, inflammation, cell proliferation and differentiation, and apoptosis. A better understanding of the relationship between *GCLC* gene polymorphisms and glutathione biosynthesis, as well as the molecular mechanisms by which this gene contributes to psoriasis, will open new scientifically based options for disease therapy and prevention targeting for glutathione metabolism. In particular, the use of L-cysteine and glycine as food supplements to restore the endogenous glutathione pool in patients with psoriasis is supported by our study results. Thus, this approach has potential in dermatological practice as a means of adjuvant therapy for psoriasis and the prevention of disease progression. Furthermore, pharmacogenetic and precision medicine approaches [88,89] would make it possible to subclassify patient groups based on environmental risk factors (e.g., cigarette smoking and alcohol abuse) and clinically significant genetic variants affecting glutathione metabolism, thus personalizing and improving the treatment and prevention of psoriasis.

## Figures and Tables

**Table 1:** Baseline and clinical characteristics of the study patients.

Characteristics	Patients with Psoriasis *n* = 474	Healthy Controls *n* = 470	*p*-Value *
Baseline characteristics			
Age, mean ± standard deviation	44.3 ± 13.6	55.3 ± 6.7	**<0.0001**
Males, *n* (%)	252 (53.2)	234 (49.8)	0.30
Females, *n* (%)	222 (46.8)	236 (50.2)	
Risk factors			
Smokers, (ever/never), *n* (%)	168 (35.4)	148 (31.5)	0.20
Alcohol abusers ^1^, *n* (%)	105 (21.2)	7 (3.2)	**<0.0001**
Location of psoriatic lesions			
Psoriatic triad	256 (54.0)	-	-
Scalp	227 (47.9)	-	
Trunk	160 (33.08)	-	-
Hands	379 (80.0)	-	-
Legs	272 (57.4)	-	-
Joints	128 (27.0)	-	-
Low back	24 (5.1)	-	-
Knees	59 (12.4)	-	-
Hips	21 (4.4)	-	-
Elbows	33 (7.0)	-	-
Fingers	60 (12.6)	-	-
Ankles	24 (5.1)	-	-
Feet/toes	23 (4.9)	-	-
Thumbs	18 (3.8)	-	-
Shoulders	11 (2.3)	-	-
Wrists	33 (7.0)	-	-
Nails	123 (25.9)	-	-
Comorbidities			
Type 2 diabetes, *n* (%)	15 (3.2)	-	-
Arterial hypertension, *n* (%)	106 (22.6)	-	-
Coronary artery disease, *n* (%)	27 (5.7)	-	-
Cerebral stroke, *n* (%)	9 (1.9)	-	-
Chronic thyroid disease, *n* (%)	7 (1.5)	-	-
Chronic renal disease, *n* (%)	30 (6.4)	-	-
Chronic gastric disease, *n* (%)	33 (7.0)	-	-
Chronic pulmonary disease, *n* (%)	7 (1.5)	-	-
Oncological disease, *n* (%)	8 (1.7)	-	-

^1^ Data on alcohol intake were available from 220 subjects of the control group. * Bold is statistically significant *p*-value.

**Table 2:** A summary of associations between *GCLC* gene polymorphisms and psoriasis risk in the entire and sex-stratified groups.

SNP ID	Minor Allele	N	Permutation *p*-Values (P_perm_) Estimated for Genetic Models of SNP–Disease Associations			
			Allelic	Additive	Dominant	Recessive
Entire groups						
rs524553	T	939	0.36	0.28	0.42	0.20
rs542914	A	941	0.18	0.23	0.67	0.11
rs648595	G	941	0.21	0.58	1.00	0.13
rs6933870	G	942	1.00	1.00	1.00	0.86
rs2397147	C	940	0.48	0.29	0.86	0.40
rs17883901	A	810	0.63	0.78	1.00	0.15
Males						
rs524553	T	485	0.38	0.43	0.50	0.20
rs542914	A	485	0.55	0.41	1.00	0.28
rs648595	G	484	**0.048**	0.23	0.86	**0.017**
rs6933870	G	485	0.25	0.13	0.32	0.09
rs2397147	C	484	0.11	0.11	0.31	**0.05**
rs17883901	A	418	1.00	1.00	1.00	0.33
Females						
rs524553	T	454	0.78	0.67	0.58	0.78
rs542914	A	456	0.59	0.32	0.59	0.48
rs648595	G	457	1.00	0.64	0.52	0.78
rs6933870	G	457	0.32	0.45	0.55	0.22
rs2397147	C	456	1.00	1.00	0.59	0.43
rs17883901	A	392	0.58	0.78	0.67	0.06

Significance of SNP–disease associations was assessed by adaptive permutations using the PLINK software, v.1.9. Bold means statistically significant *p*-values (P_perm_).

**Table 3:** Genotype and allele frequencies of the *GCLC* gene in healthy controls and patients with psoriasis *.

SNP	Genotype/ Allele	Healthy Controls *n* (%) ^1^	Patients with Psoriasis *n* (%) ^1^	OR ^2^ (95% CI)	*P*_perm_^3^
Entire groups					
rs524553	C/C	273 (58.3)	285 (60.5)	0.67 (0.34–1.30)	0.20 ^R^
	C/T	173 (37.0)	171 (36.3)		
	T/T	22 (4.7)	15 (3.2)		
	T	217 (23.2)	201 (21.3)	0.90 (0.72–1.12)	0.36
rs542914	C/C	168 (35.8)	174 (36.9)	0.75 (0.52–1.08)	0.11 ^R^
	C/A	227 (48.4)	240 (50.9)		
	A/A	74 (15.8)	58 (12.3)		
	A	375 (40.0)	356 (37.7)	0.91 (0.76–1.09)	0.18
rs648595	T/T	147 (31.4)	144 (30.4)	0.75 (0.54–1.05)	0.13 ^R^
	T/G	225 (48.1)	252 (53.3)		
	G/G	96 (20.5)	77 (16.3)		
	G	417 (44.6)	406 (42.9)	0.94 (0.78–1.12)	0.21
rs6933870	C/C	160 (34.0)	163 (34.5)	0.93 (0.65–1.33)	0.86 ^R^
	C/G	237 (50.4)	240 (50.9)		
	G/G	73 (15.5)	69 (14.6)		
	G	383 (40.7)	378 (40.0)	0.97 (0.81–1.17)	0.99
rs2397147	T/T	183 (39.2)	198 (41.9)	0.90 (0.74–1.09)	0.29 ^A^
	T/C	231 (49.5)	230 (48.6)		
	C/C	53 (11.3)	45 (9.5)		
	C	337 (36.1)	320 (33.8)	0.91 (0.75–1.09)	0.48
rs17883901	G/G	334 (89.1)	388 (89.2)	0.43 (0.11–1.72)	0.15 ^R^
	G/A	35 (9.3)	44 (10.1)		
	A/A	6 (1.6)	3 (0.7)		
	A	47 (6.3)	50 (5.7)	0.91 (0.60–1.38)	0.63
Males					
rs524553	C/C	137 (58.5)	152 (60.6)	0.56 (0.23–1.38)	0.20 ^R^
	C/T	84 (35.9)	91 (36.2)		
	T/T	13 (5.6)	8 (3.2)		
	T	110 (23.5)	107 (21.3)	0.88 (0.65–1.19)	0.38
rs542914	C/C	81 (34.6)	87 (34.7)	0.75 (0.44–1.26)	0.28 ^R^
	C/A	117 (50.0)	134 (53.4)		
	A/A	36 (15.4)	30 (11.9)		
	A	189 (40.4)	194 (38.6)	0.93 (0.72–1.20)	0.55
rs648595	T/T	71 (30.5)	78 (31.1)	**0.56 (0.35–0.90)**	**0.017 ^R^**
	T/G	110 (47.2)	138 (55.0)		
	G/G	52 (22.3)	35 (13.9)		
	G	214 (45.9)	208 (41.4)	0.83 (0.65–1.07)	**0.048**
rs6933870	C/C	73 (31.2)	87 (34.7)	0.64 (0.38–1.06)	0.09 ^R^
	C/G	120 (51.3)	134 (53.4)		
	G/G	41 (17.5)	30 (11.9)		
	G	202 (43.2)	194 (38.6)	0.83 (0.64–1.07)	0.25
rs2397147	T/T	85 (36.5)	101 (40.2)	**0.54 (0.30–0.98)**	**0.05 ^R^**
	T/C	116 (49.8)	130 (51.8)		
	C/C	32 (13.7)	20 (8.0)		
	C	180 (38.6)	170 (33.9)	0.81 (0.63–1.06)	0.11
rs17883901	G/G	167 (89.3)	204 (88.3)	0.54 (0.09–3.24)	0.33 ^R^
	G/A	17 (9.1)	25 (10.8)		
	A/A	3 (1.6)	2 (0.9)		
	A	23 (6.1)	29 (6.3)	1.02 (0.58–1.80	0.99
Females					
rs524553	C/C	136 (58.1)	133 (60.5)	0.91 (0.62–1.32)	0.58 ^D^
	C/T	89 (38.0)	80 (36.4)		
	T/T	9 (3.8)	7 (3.2)		
	T	107 (22.9)	94 (21.4)	0.92 (0.67–1.25)	0.78
rs542914	C/C	87 (37.0)	87 (39.4)	0.88 (0.68–1.15)	0.32 ^A^
	C/A	110 (46.8)	106 (48)		
	A/A	38 (16.2)	28 (12.7)		
	A	186 (39.6)	162 (36.7)	0.88 (0.68–1.15)	0.59
rs648595	T/T	76 (32.3)	66 (29.7)	1.13 (0.76–1.68)	0.52 ^D^
	T/G	115 (48.9)	114 (51.4)		
	G/G	44 (18.7)	42 (18.9)		
	G	203 (43.2)	198 (44.6)	1.06 (0.82–1.37)	0.99
rs6933870	C/C	87 (36.9)	76 (34.4)	1.37 (0.82–2.27)	0.22 ^R^
	C/G	117 (49.6)	106 (48.0)		
	G/G	32 (13.6)	39 (17.6)		
	G	181 (38.3)	184 (41.6)	1.15 (0.88–1.49)	0.32
rs2397147	T/T	98 (41.9)	97 (43.7)	1.29 (0.70–2.37)	0.43 ^R^
	T/C	115 (49.1)	100 (45)		
	C/C	21 (9.0)	25 (11.3)		
	C	157 (33.5)	150 (33.8)	1.01 (0.77–1.33)	0.99
rs17883901	G/G	167 (88.8)	184 (90.2)	0.30 (0.03–2.95)	0.06 ^R^
	G/A	18 (9.6)	19 (9.3)		
	A/A	3 (1.6)	1 (0.5)		
	A	24 (6.4)	21 (5.1)	0.80 (0.44–1.45)	0.58

* The table shows the best genetic models for SNP–disease associations. ^1^ Absolute number and percentage of individuals/chromosomes with a particular genotype/allele. ^2^ Odds ratio with 95% confidence intervals (crude analysis) estimated for the best association model. ^3^ *p*-value estimated for the best association model via adaptive permutations. Superscripts denote SNP association models: ^R^, recessive; ^D^, dominant; ^A^, additive. Bold depicts statistically significant *p*-values and odds ratios.

**Table 4:** Haplotypes of the *GCLC* gene and their association with psoriasis risk in the entire and sex-stratified groups.

Haplotypes	SNP						Patients with Psoriasis	Healthy Controls	Chi Square	*p*-Value
	rs524553	rs542914	rs648595	rs6933870	rs2397147	rs17883901				
Entire groups										
H1	C	C	T	C	T	G	0.482	0.463	0.635	0.426
H2	T	A	G	G	C	G	0.154	0.162	0.192	0.661
H3	C	A	G	G	C	G	0.121	0.128	0.186	0.666
H4	C	C	G	G	T	G	0.056	0.043	1.681	0.195
H5	C	A	T	C	T	G	0.043	0.042	0.017	0.898
H6	C	C	G	C	T	G	0.032	0.027	0.422	0.516
H7	C	C	T	C	T	A	0.019	0.026	0.940	0.332
H8	T	A	G	C	T	G	0.017	0.027	2.247	0.134
H9	T	A	G	G	C	A	0.023	0.020	0.133	0.715
H10	C	C	T	G	C	G	0.018	0.018	0.016	0.900
H11	C	A	G	G	C	A	0.010	0.013	0.301	0.583
H12	-	-	-	-	-	-	-	-	-	-
Males										
H1	C	C	T	C	T	G	0.495	0.457	1.407	0.236
H2	T	A	G	G	C	G	0.160	0.175	0.357	0.550
H3	C	A	G	G	C	G	0.115	0.136	0.989	0.320
H4	C	C	G	G	T	G	0.045	0.043	0.017	0.896
H5	C	A	T	C	T	G	0.048	0.030	2.040	0.153
H6	C	C	G	C	T	G	0.026	0.024	0.040	0.842
H7	C	C	T	C	T	A	0.020	0.024	0.223	0.637
H8	T	A	G	C	T	G	0.016	0.022	0.456	0.499
H9	T	A	G	G	C	A	0.025	0.018	0.604	0.437
H10	C	C	T	G	C	G	0.015	0.023	0.839	0.359
H11	C	A	G	G	C	A	0.013	0.014	0.030	0.863
H12	-	-	-	-	-	-	-	-	-	-
Females										
H1	C	C	T	C	T	G	0.463	0.464	0.001	0.981
H2	T	A	G	G	C	G	0.150	0.158	0.109	0.741
H3	C	A	G	G	C	G	0.130	0.119	0.243	0.622
H4	C	C	G	G	T	G	0.069	0.045	2.445	0.118
H5	C	A	T	C	T	G	0.037	0.052	1.155	0.283
H6	C	C	G	C	T	G	0.034	0.027	0.418	0.518
H7	C	C	T	C	T	A	0.023	0.028	0.321	0.571
H8	T	A	G	C	T	G	0.018	0.031	1.511	0.219
H9	T	A	G	G	C	A	0.017	0.017	0.010	0.919
H10	C	C	T	G	C	G	0.021	0.013	0.761	0.383
H11	C	A	G	G	C	A	-	-	-	-
H12	T	C	G	G	C	G	0.010	0.010	0.001	0.983

Estimation of haplotype frequencies and significance of haplotype–disease associations was conducted using the Haploview software, v.4.2.

**Table 5:** *GCLC* genotype combinations showed associations with psoriasis risk.

Genotype Combination	Patients		Controls		*p*-Value	OR (95% CI) ^3^
	*n* ^1^	% ^2^	*n* ^1^	% ^2^		
Entire groups						
rs542914-C/C × rs648595-G/T	55	11.7	35	7.5	**0.03**	1.63 (1.04–2.54)
rs648595-G/G × rs6933870-C/G	13	2.8	28	6.0	**0.016**	0.45 (0.23–0.87)
Males						
rs524553-C/C × rs648595-G/G	7	2.8	18	7.7	0.025	0.36 (0.15–0.85)
rs524553-C/C × rs6933870-G/G	6	2.4	15	6.4	0.05	0.37 (0.15–0.95)
rs542914-A/A × rs648595-G/G	19	7.6	31	13.3	0.038	0.54 (0.30–0.98)
rs648595-G/G × rs17883901-G/G	21	9.1	33	17.7	0.009	0.47 (0.26–0.84)
rs6933870-G/G × rs2397147-C/C	20	8.0	32	13.7	0.042	0.55 (0.30–0.99)
rs6933870-G/G × rs17883901-G/G	19	8.2	27	14.4	0.044	0.53 (0.29–0.99)
rs2397147-C/C × rs17883901-G/G	11	4.8	21	11.2	**0.014**	0.40 (0.19–0.85)
Females						
rs6933870-G/G × rs17883901-G/G	32	15.8	17	9.0	**0.045**	1.88 (1.01–3.52)

^1^ Absolute number of individuals with particular genotype combination (minor alleles in genotypes are underlined). ^2^ Percentage of individuals with particular genotype combination. ^3^ OR, odds ratio; CI, confidence interval. Bold is statistically significant *p*-value after an adjustment for FDR of 0.05 (https://tools.carbocation.com/FDR, accessed on 2 April 2023).

**Table 6:** A summary of associations between *GCLC* gene polymorphisms and psoriasis risk in groups stratified by cigarette smoking and alcohol abuse.

SNP ID	Minor Allele	Permutation *p*-Values (P_perm_) Estimated for Genetic Models of SNP–Disease Associations									
		N	Genetic Models				N	Genetic Models			
			Allelic	Additive	Dominant	Recessive		Allelic	Additive	Dominant	Recessive
		Smokers					Non-smokers				
rs524553	T	315	1.00	0.52	0.63	0.64	624	0.46	0.43	0.86	0.34
rs542914	A	315	0.86	0.55	0.67	0.67	626	0.21	0.59	0.86	0.10
rs648595	G	316	0.12	0.44	0.52	**0.049**	625	0.86	0.78	1.00	0.52
rs6933870	G	315	0.65	0.52	0.86	0.59	627	1.00	1.00	0.86	0.67
rs2397147	C	315	0.67	0.33	0.48	0.25	625	0.86	0.46	0.39	0.73
rs17883901	A	275	0.24	0.16	0.09	1.00	535	0.18	0.11	0.18	**0.002**
		Alcohol abusers					Non-drinkers				
rs524553	T	110	0.26	0.09	0.10	NA	580	0.24	0.08	0.20	0.15
rs542914	A	112	0.11	0.053	0.06	NA	579	**0.034**	**0.026**	0.16	**0.015**
rs648595	G	112	0.33	0.19	0.58	NA	580	**0.05**	**0.04**	0.26	**0.03**
rs6933870	G	111	0.18	0.11	0.23	NA	581	0.29	0.14	0.18	0.27
rs2397147	C	112	0.19	0.22	0.14	NA	579	0.20	0.09	0.14	0.25
rs17883901	A	98	0.79	NA	NA	NA	498	0.55	0.48	0.67	0.09

Significance of SNP–disease associations was assessed by adaptive permutations using the PLINK software, v.1.9. NA, not available. Bold means statistically significant *p*-values (P_perm_).

**Table 7:** Replication of associations between the studied polymorphisms of the *GCLC* gene and psoriasis risk in a population of the UK Biobank ^1^.

Psoriasis Phenotype ^2^	Variant	Eff, Allele	Beta	OR Beta	*p*-Value	MAF	HWE
psoriasis	rs524553	T	0.00030445	1.03	0.24054	0.248703	0.8257
L40 Psoriasis	rs524553	T	0.00014144	1.03	0.43139	0.248703	0.8257
psoriasis	rs542914	A	0.00031437	1.03	0.16739	0.409665	0.7591
L40 Psoriasis	rs542914	A	0.00018358	1.03	0.24466	0.409665	0.7591
psoriasis	rs648595	G	0.00034186	1.03	0.12131	0.485677	0.2804
L40 Psoriasis	rs648595	G	0.00019125	1.04	0.21101	0.485677	0.2804
psoriasis	rs6933870	G	0.00041555	1.04	0.062535	0.478105	0.1793
L40 Psoriasis	rs6933870	G	0.00015391	1.03	0.3195	0.478105	0.1793
psoriasis	rs2397147	C	0.00043391	1.04	0.057101	0.407803	0.6808
L40 Psoriasis	rs2397147	C	0.000164	1.03	0.29943	0.407803	0.6808
psoriasis	rs17883901	G	−0.0001631	0.986	0.68281	0.0837	0.05018
L40 Psoriasis	rs17883901	G	−0.0002265	0.959	0.4129	0.0837	0.05018

^1^ The calculations were obtained from the Gene ATLAS website (http://geneatlas.roslin.ed.ac.uk/), accessed on 28 April 2023. ^2^ “Psoriasis” phenotype investigated in a cohort of 5175 cases and 447,089 controls); “L40 Psoriasis” phenotype investigated in a cohort of 2437 cases and 449,827 controls. MAF, minor allele frequency; HWE, Hardy–Weinberg equilibrium *p*-value.

**Table 8:** Polymorphisms of the *GCLC* gene showed significant associations (*p* ≤ 0.05) with the risk of psoriasis in a population of the UK Biobank.

N	Variant	Position	Eff, Allele	Trait	Beta	*p*-Value	MAF
Psoriasis phenotype: “psoriasis” (5175 cases and 447,089 controls)							
1	rs183555084	53463377	A	psoriasis	0.0054328	0.00048311	0.005415
2	rs536001584	53491157	A	psoriasis	0.0069889	0.0037087	0.002296
3	rs78863400	53507843	G	psoriasis	0.0020045	0.0049101	0.0245
4	rs114919458	53478492	A	psoriasis	0.0020638	0.0077221	0.020874
5	rs77162334	53473387	A	psoriasis	0.0015616	0.0084049	0.036438
6	rs547541077	53524639	A	psoriasis	0.0083841	0.011327	0.001201
7	rs55661362	53463674	G	psoriasis	0.0026909	0.012278	0.011041
8	rs78331008	53489705	G	psoriasis	0.0014586	0.014341	0.036042
9	rs115558853	53325654	C	psoriasis	−0.0019831	0.016188	0.018765
10	rs6902510	53493460	T	psoriasis	−0.00052994	0.019054	0.405458
11	rs62398116	53405203	G	psoriasis	−0.0008575	0.019471	0.110262
12	rs189491343	53341496	G	psoriasis	−0.0019418	0.020189	0.01847
13	rs7762921	53319569	T	psoriasis	−0.00065758	0.021591	0.1828
14	rs62398159	53490625	A	psoriasis	−0.00051684	0.022213	0.406839
15	rs56013020	53390696	A	psoriasis	0.00082793	0.022789	0.103296
16	rs7739121	53510423	C	psoriasis	−0.00049679	0.024997	0.467815
17	rs72944719	53358473	G	psoriasis	−0.0010805	0.025673	0.05524
18	rs7761225	53315323	C	psoriasis	−0.00064261	0.025687	0.179565
19	rs6458936	53314296	G	psoriasis	−0.00064298	0.025697	0.179334
20	rs1914707	53311047	G	psoriasis	−0.00063643	0.026486	0.181629
21	rs563831	53327107	G	psoriasis	0.00063512	0.026486	0.183707
22	rs4715409	53511015	T	psoriasis	−0.00049168	0.02667	0.467022
23	rs1518511	53313237	C	psoriasis	−0.00063702	0.027149	0.179343
24	rs6908614	53501678	T	psoriasis	−0.00048943	0.027196	0.462606
25	rs642103	53323152	G	psoriasis	−0.00062689	0.028507	0.18174
26	rs1914706	53311463	T	psoriasis	−0.00062752	0.028627	0.181766
27	rs72943672	53399516	T	psoriasis	−0.00074715	0.028945	0.1182
28	rs6933919	53313748	G	psoriasis	−0.00062901	0.029059	0.179555
29	rs4712030	53317469	A	psoriasis	−0.00062437	0.029102	0.181758
30	rs1467408	53351289	A	psoriasis	−0.00052426	0.029222	0.361091
31	rs9382209	53311804	G	psoriasis	−0.00062389	0.02952	0.18191
32	rs149644917	53519358	A	psoriasis	−0.010751	0.029585	0.000499
33	rs1401155	53312629	C	psoriasis	−0.00062709	0.029593	0.17955
34	rs9357769	53508264	C	psoriasis	0.00048131	0.029829	0.4664
35	rs6908786	53494357	A	psoriasis	−0.00047818	0.03092	0.466556
36	rs587178	53325255	T	psoriasis	0.00061535	0.031491	0.182191
37	rs6901352	53500138	C	psoriasis	−0.0004754	0.031615	0.466514
38	rs6908860	53494615	T	psoriasis	−0.00047638	0.031652	0.464814
39	rs681682	53440021	C	psoriasis	−0.0072738	0.032871	0.001361
40	rs543473	53439524	T	psoriasis	−0.0072796	0.032941	0.001359
41	rs681585	53439958	G	psoriasis	−0.0072742	0.033023	0.00136
42	rs9474608	53505134	A	psoriasis	−0.00047139	0.033072	0.466612
43	rs681635	53439987	A	psoriasis	−0.0072632	0.033272	0.001359
44	rs2397146	53360119	A	psoriasis	−0.00053256	0.033642	0.273716
45	rs607285	53326491	T	psoriasis	0.00060766	0.033745	0.182155
46	rs62416866	53398370	A	psoriasis	−0.00077679	0.033936	0.100838
47	rs742528	53360191	A	psoriasis	−0.00052981	0.034548	0.273993
48	rs623928	53335695	T	psoriasis	0.00061135	0.034551	0.180506
49	rs629162	53326283	G	psoriasis	0.00060422	0.034685	0.182369
50	rs676637	53335353	C	psoriasis	0.00061072	0.03473	0.180538
51	rs624432	53335555	G	psoriasis	0.00061046	0.034804	0.180555
52	rs642625	53333732	T	psoriasis	0.00061027	0.034833	0.180511
53	rs618033	53339289	T	psoriasis	0.00061046	0.034957	0.180357
54	rs600722	53332887	T	psoriasis	0.00060973	0.034961	0.180513
55	rs631783	53338531	A	psoriasis	0.00060876	0.035396	0.180454
56	rs619955	53338845	T	psoriasis	0.00060877	0.035396	0.180457
57	rs485371	53341627	T	psoriasis	0.00060874	0.035527	0.180356
58	rs12196344	53457292	A	psoriasis	−0.00048763	0.036061	0.404087
59	rs9367538	53506487	G	psoriasis	−0.00046273	0.036479	0.466245
60	rs7764361	53492467	C	psoriasis	0.00046427	0.037421	0.456163
61	rs663087	53342704	T	psoriasis	0.00060223	0.037659	0.180217
62	rs646403	53347484	T	psoriasis	0.00059431	0.040381	0.180136
63	rs12194171	53464937	C	psoriasis	0.00046011	0.041523	0.3968
64	rs11756739	53316777	A	psoriasis	0.0029885	0.04429	0.006094
65	rs4712031	53320273	G	psoriasis	−0.00056517	0.04448	0.190022
66	rs2092421	53473076	A	psoriasis	−0.00045072	0.045589	0.398208
67	rs4269374	53461179	G	psoriasis	−0.00044872	0.04647	0.397012
68	rs9349679	53470507	A	psoriasis	−0.00044669	0.047497	0.39642
69	rs34997452	53518439	T	psoriasis	−0.0027868	0.047543	0.006643
70	rs10807461	53472150	T	psoriasis	−0.00044608	0.047762	0.398057
71	rs738472	53477038	C	psoriasis	−0.00045789	0.048043	0.353182
72	rs6458946	53472830	T	psoriasis	−0.00044442	0.048672	0.397982
73	rs114749455	53489865	G	psoriasis	0.0022206	0.048751	0.0103
74	rs2143399	53461749	A	psoriasis	−0.00044292	0.049341	0.397029
75	rs74357476	53476523	T	psoriasis	0.0014009	0.050596	0.025311
Psoriasis phenotype: “L40 Psoriasis” (2437 cases and 449,827 controls)							
1	rs185956124	53496212	C	L40 Psoriasis	0.0026856	0.0036274	0.00747649
2	rs547541077	53524639	A	L40 Psoriasis	0.0065265	0.0044446	0.00120121
3	rs189622943	53509408	T	L40 Psoriasis	0.0035101	0.0095446	0.00341133
4	rs183043870	53509634	G	L40 Psoriasis	0.0035128	0.0095673	0.00341141
5	rs78735978	53360036	C	L40 Psoriasis	0.0012576	0.015913	0.0231714
6	rs41271287	53370147	T	L40 Psoriasis	0.0011902	0.018795	0.0236652
7	rs17215384	53510321	T	L40 Psoriasis	0.00039365	0.02118	0.28084
8	rs77516417	53373662	A	L40 Psoriasis	−0.001175	0.021204	0.02313
9	rs574202	53481989	G	L40 Psoriasis	0.00035417	0.021427	0.489829
10	rs12661112	53486714	A	L40 Psoriasis	0.00037194	0.021838	0.343991
11	rs563699	53479410	C	L40 Psoriasis	0.00035124	0.022359	0.490659
12	rs558026	53478773	A	L40 Psoriasis	0.00035803	0.022979	0.392597
13	rs583513	53477688	T	L40 Psoriasis	0.00034605	0.024098	0.491525
14	rs7759126	53484485	C	L40 Psoriasis	0.00035645	0.028105	0.343339
15	rs12665537	53509452	G	L40 Psoriasis	0.00035343	0.030008	0.33107
16	rs67228890	53511814	G	L40 Psoriasis	0.00034794	0.034841	0.327456
17	rs74449072	53521238	G	L40 Psoriasis	0.00061918	0.039019	0.0749875
18	rs7764361	53492467	C	L40 Psoriasis	0.00031881	0.039179	0.456163
19	rs9382225	53511696	T	L40 Psoriasis	−0.00033714	0.039962	0.328914
20	rs5020412	53349885	C	L40 Psoriasis	0.00084548	0.041197	0.0354
21	rs4715412	53511836	T	L40 Psoriasis	−0.00033138	0.044253	0.328611

## Data Availability

Data supporting reported results are available upon request.
